# Dissection of pro-tumoral macrophage subtypes and immunosuppressive cells participating in M2 polarization

**DOI:** 10.1007/s00011-024-01907-3

**Published:** 2024-06-27

**Authors:** Onurcan Sezginer, Nese Unver

**Affiliations:** Department of Basic Oncology, Cancer Institute, Hacettepe University, Sihhiye, Ankara, 06100 Türkiye

**Keywords:** Tumor-associated macrophage, M2 macrophage, Macrophage polarization, Immunosuppressive niche, Immunotherapy

## Abstract

Alternatively activated macrophage (M2) polarization can result in one of four subtypes based on cytokines and signaling pathways associated with macrophage activation: M2a, M2b, M2c, and M2d macrophages. The majority of M2 subtypes are anti-inflammatory and pro-angiogenic, secreting growth factors (VEGF, PDGF) and matrix metalloproteinases (MMP2, MMP9) which boost tumor growth, metastasis, and invasion. M2-polarized macrophages are associated with immune suppressor cells harboring Myeloid derived suppressor cells, Regulatory T cells (Tregs), Regulatory B cells as well as alternatively activated (N2) neutrophils. Treg cells selectively support the metabolic stability, mitochondrial integrity, and survival rate of M2-like TAMs in an indirect environment. Also, the contribution of Breg cells influences macrophage polarization towards the M2 direction. TAM is activated when TAN levels in the tumor microenvironment are insufficient or vice versa, suggesting that macrophage and its polarization are fine-tuned. Understanding the functions of immune suppressive cells, mediators, and signaling pathways involved with M2 polarization will allow us to identify potential strategies for targeting the TAM repolarization phenotype for innovative immunotherapy approaches. In this review, we have highlighted the critical factors for M2 macrophage polarization, differential cytokine/chemokine profiles of M1 and M2 macrophage subtypes, and other immune cells’ impact on the polarization within the immunosuppressive niche.

## Highlights

M2 macrophages are targetable immune cells for immunotherapy strategies.

M2 type macrophages are more heterogeneous populations than M1 macrophages.

M2 polarization is influenced by Tregs, Bregs, MDSCs, and N2 neutrophils.

**Graphical Abstract:**

## Graphical abstract

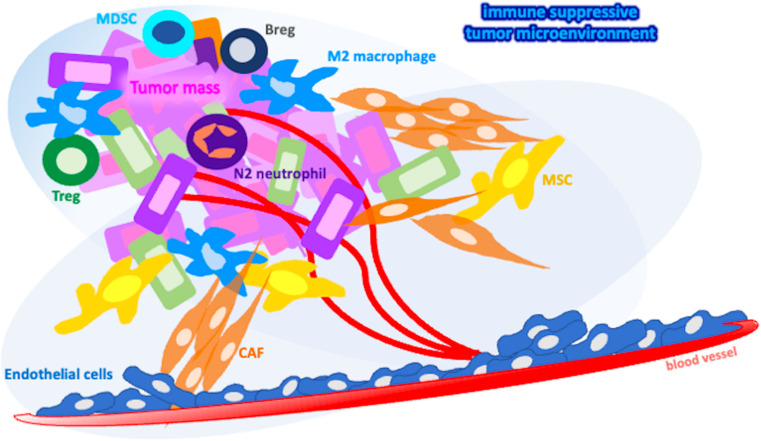

## Introduction

Tumor niche is a multifaceted complex that is host to a wide range of cellular and non-cellular actors [1]. Among these actors, due to their remarkable plasticity and range of functions, macrophages hold an important place. Albeit having anti-tumoral properties in early stages of tumor progression, macrophages termed as tumor-associated macrophages (TAMs) residing in the tumor niche have been associated with multiple types of malignancies, including tumorigenesis, vascularization, invasion and metastasis of tumor and drug resistance [2]. The functional differences of macrophages are directly linked with their polarization.

Macrophage polarization results in primarily two distinct subtypes, namely M1 or M2 macrophages. Among these subtypes, M2 macrophages are the primary culprit in supporting tumor growth and have been the target of anti-cancer therapies [3]. M2 macrophages have been reported to induce tumor progression through secretion of a wide variety of proliferation inducing and immunosuppressive cytokines and chemokines. In addition to their secretory profiles, they have been reported to have cell-cell interactions that hinder immune response against tumor cells. Many cells residing in the TME induce M2 like TAM polarization, including immunosuppressive cells and tumor associated stromal cells [4–6]. M2-like polarization of TAMs should be understood well for the generation of novel therapeutic strategies and improvement of foregoing therapeutic approaches.

This review discusses participation of immune suppressor cells in acquiring the M2 phenotype of macrophages at the cellular and molecular levels, in addition to underlining the importance of macrophage polarization in the tumor microenvironment. We have also emphasized the significance of secretory profiles and immunotherapeutic targeting of both M2 macrophage subtypes and their potential roles in the immunosuppressive niche.

## Tumor associated macrophages (TAMs) in tumor microenvironment

**Fig. 1:**
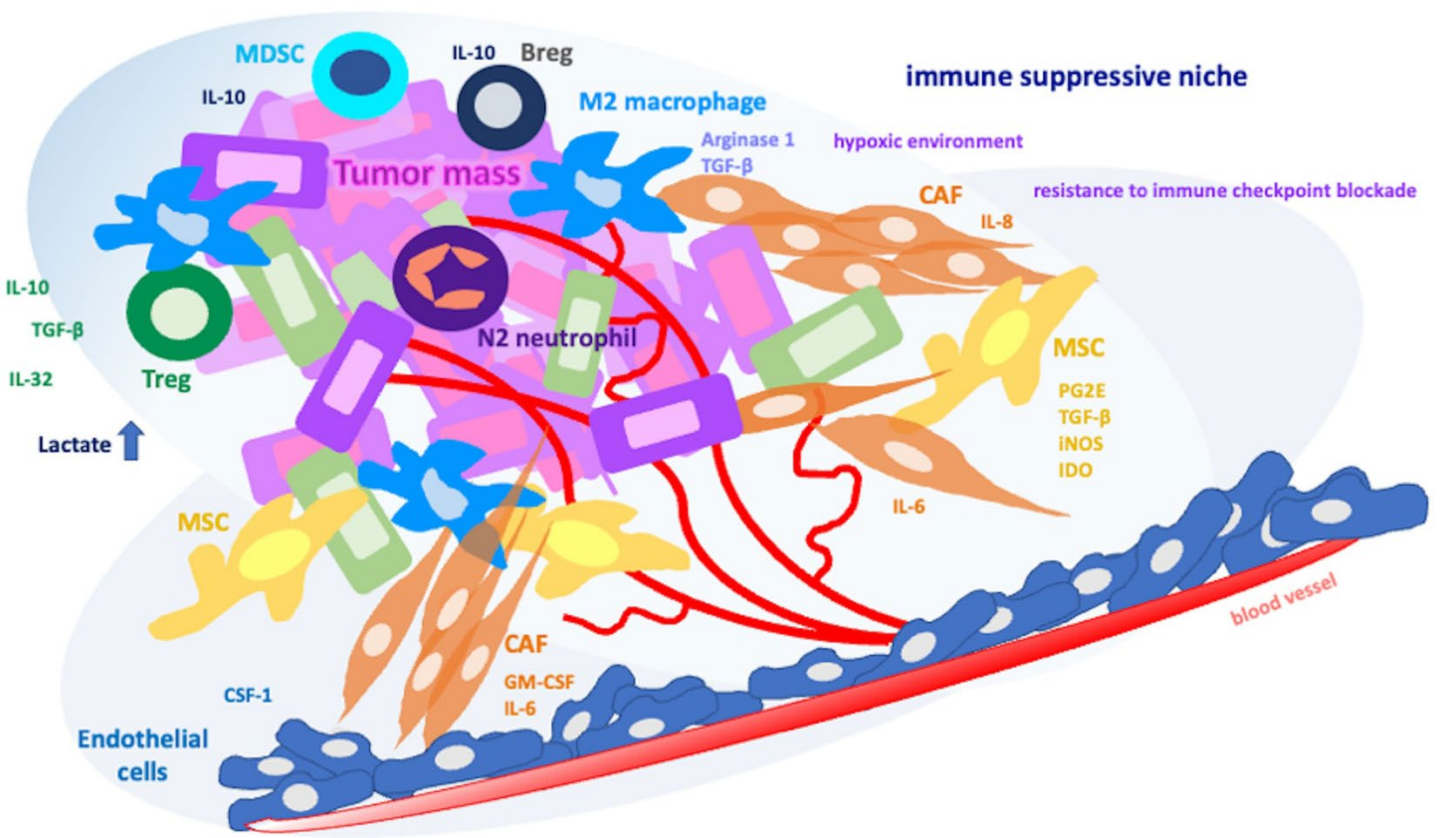
Immunosuppressive cells in cancer leading to the pro-tumoral (M2) macrophage polarization

The tumor microenvironment (TME) is a dynamic entity composed of cellular and non-cellular components that induce tumor growth and progression. The non-cellular components of the TME consists of the extracellular matrix (ECM), exosomes as well as cytokines and chemokines secreted by cellular components of the TME, which contain a vast variety of cells such as endothelial cells, fibroblasts, adipocytes, neuroendocrine cells, and immune cells (Fig. 1) [7].

Tumor cells attract circulating monocytes to the TME in response to several stimuli such as VEGF, CSF-1, TGF-β, GM-CSF, IL-6, IL-10, and chemokines. The differentiation and polarization of arriving monocytes into TAMs is influenced by cytokines, chemokines, and growth factors released by cellular components of TME (Fig. 1) [8].

The presence of TAMs is often associated with poor prognosis of patients with several types of cancer. Poor prognosis in gastric cancer [9], breast cancer [10], cervical cancer [11] are associated with multiple characteristics of TAMs, such as promoting angiogenesis, tumor migration and metastasis, and immune regulation.

### TAMs, a subpopulation found in intratumoral hypoxic zones, play an important role in facilitating solid tumor growth and metastasis

Hypoxia, one of the hallmarks of TME, can affect the TAMs function as TAMs differentially express hypoxia-inducible factor-1α (HIF1α) and HIF2α, which are the primary factors expressed in response to hypoxia [12, 13].Through these factors, which leads to upregulation of angiogenic growth factors VEGF, fibroblast growth factor 2 (FGF2), platelet derived growth factor (PDGF), TAM driven vessel formation can occur in TME (Fig. 1).

Endothelial degradation primarily through matrix metalloproteinases MMP2, MMP7 and MMP9 is not only required for angiogenesis, but also promotes tumor migration and metastasis. Metalloproteinases, as well as serine proteases and cathepsins secreted by TAMs can degrade matrix membranes of endothelial cells surrounding tumor tissue to induce tumor dissociation, migration, and metastasis [14]. Thus, TAMs can directly, through release of angiogenic factors, or indirectly via endothelial degradation and upregulation of angiogenic modulators, achieve proangiogenic response to hypoxic conditions of the TME.

### TAMs have been shown to serve an important role in epithelial-mesenchymal transition (EMT) in addition to their role in tumor vascularization

Mesenchymal cancer cells can induce TAM-like phenotype in macrophages via secretion of GM-CSF which creates a positive feedback loop between TAMs and EMT [15]. In addition to aforementioned factors that induce EMT, IL-6, IL-8 and IL-10 secreted by TAMs also promote mesenchymal transition (Fig. 1).

TGF-β, a factor released by TAMs that plays an important role in EMT, can stimulate proliferation in later stages of tumor growth [16]. It leads to phosphorylation of SMAD2 and SMAD3 which combine with SMAD4 to form SMAD trimeric complex that translocates to the nucleus and induces transcription of EMT related genes δEF1 and SIP1. δEF1 and SIP1 in turn inhibits the expression of the alternative splicing factor ESRP to help cells go through EMT induced by TGF-β [17]. Thus, TGF-β has emerged to exert EMT triggering characteristics on tumor cells, and pro-tumoral TAM phenotype coincides with TGF-β [16].

Tumor necrosis factor-α (TNF-α) is another important cytokine that induces EMT. Endogenous secretion of TNF-α by TAMs and tumor cells induce EMT by inhibiting epithelial marker E-cadherin transcription and upregulating mesenchymal markers N-cadherin, fibronectin, and vimentin expression. TNF-α also induces MMP-9 expression which enhances tumor cells’ ability to migrate and invade other tissues [18]. Along with their individual abilities to induce EMT, TNF-α and TGF-β can also work in concordance with one another to induce EMT. For example, in cervical cancer, TNF-α and TGF-β are shown to be working cooperatively to induce EMT and tumor stemness through NF-κB/Twist axis [19].

TAM-mediated IL-6 overexpression has been found to drive EMT in a β-catenin dependent manner [20]. Another cytokine, IL-8 acts to inhibit PI3K-Akt signaling and E-cadherin expression in tumor cells, which induces the acquisition of mesenchymal phenotype in tumor cells [21]. TAMs are also reported to induce EMT through Toll-like receptor 4 (TLR4)/IL-10 signaling by inducing mesenchymal markers Vimentin and Snail expression while simultaneously downregulating E-cadherin [22].

### Tumor-associated macrophages modulate tumorigenicity of cancer stem cells

After acquiring a mesenchymal phenotype, cancer cells can enter a stem cell like state to become cancer stem cells (CSCs) with increased tumorigenic potential and chemoresistance [23]. CSCs as a concept can provide a better understanding of neoplastic progression, dissemination, and relapse of cancer [24]. A positive feedback loop occurs between CSCs and TAMs as CSCs through secretion of chemotactic factors CCL2, CCL3, CCL5, CCL8 and CXCL12 can recruit monocytes to TME. Recruited monocytes then gain a pro-tumoral phenotype through cytokines IL-4, IL-6, IL-10, IL13 and TGF-β; again, secreted by CSCs. Pro-tumoral TAMs, in addition to induce EMT as mentioned before, can sustain CSCs’ niche through secretion of cytokines (IL-6, IL-1β, TNF-α and TGF-β) and chemokines (CCL2, CCL5 and CCL8) and inducing the expression of the transcription factor NANOG, SOX2, OCT3 and OCT4 [25]. In addition to secretory mechanisms, cell-to-cell interaction has been shown to trigger pathways critical for CSCs. For example, SHH pathway in colorectal cancer, as well as TGF-β/SMAD/NANOG pathway in pancreatic cancer have been reported to be upregulated by physical interactions between TAMs and CSCs [24, 25].

Among the factors that induce cancer stemness, glycoprotein NMB (GPNMB) is one of the most crucial proteins. GPNMB in TME is mainly expressed by TAMs, can be cleaved into a soluble form by ADAM10 which then binds to CD44 receptor in cancer cells to induce release several chemokines (CXCL1, CXCL2, CCL2, CCL5 and CCL7) as well as cytokines (IL-6, IL-11, and IL-33) [26]. IL-33 can bind to its receptor in TAMs, IL-1RL1, to induce TGF-β secretion by TAMs; while it can also bind to IL-1RL1 in other cancer cells to increase the population of CSCs through inducing the expression of stem cell genes (NANOG, NOTCH3, OCT3 and OCT4) as well as increase chemoresistance of cancer cells [27].

### TAMs can both promote and suppress immunological responses against tumor cells

TAMs can induce immune response through activating CD8^+^ T cells and natural killer (NK) cells, phagocytosis of tumor cells, and release of nitric oxide and reactive oxygen species [28]. Furthermore, TAMs can stimulate the activation of dendritic cells (DCs), which in turn can present tumor antigens to CD8^+^ T cells and promote host immune response against tumors [29]. However, TAMs immunosuppressive features are far more common than its immuno-stimulant effects as they are known to secrete anti-inflammatory cytokines, primarily IL-10 and TGF-β [30].

In addition to cytokine production, TAMs can limit T cell activity by decreasing T cell receptor function through suppression of CD3-ζ chain expression via oxidative stress [31]. T regulatory cells (Tregs) can also be recruited to TME by TAMs via CCL22 secretion, which also serves to prevent T cell activation.

TAMs can upregulate PD-1 and PD-L1 expression, which leads to T cell exhaustion and dysfunction [32]. TAMs are capable of inducing T cell exhaustion by preventing DC maturation. If not properly matured, DCs will have diminished expression of costimulatory molecules CD80 and CD86, and substantial expression of immunosuppressive PD-L1 expression, resulting in anergic T cells and impaired anti-tumoral host immune responses [33].

### M1 and M2 macrophage subtypes are critical contributors to the tumor microenvironment

**Fig. 2:**
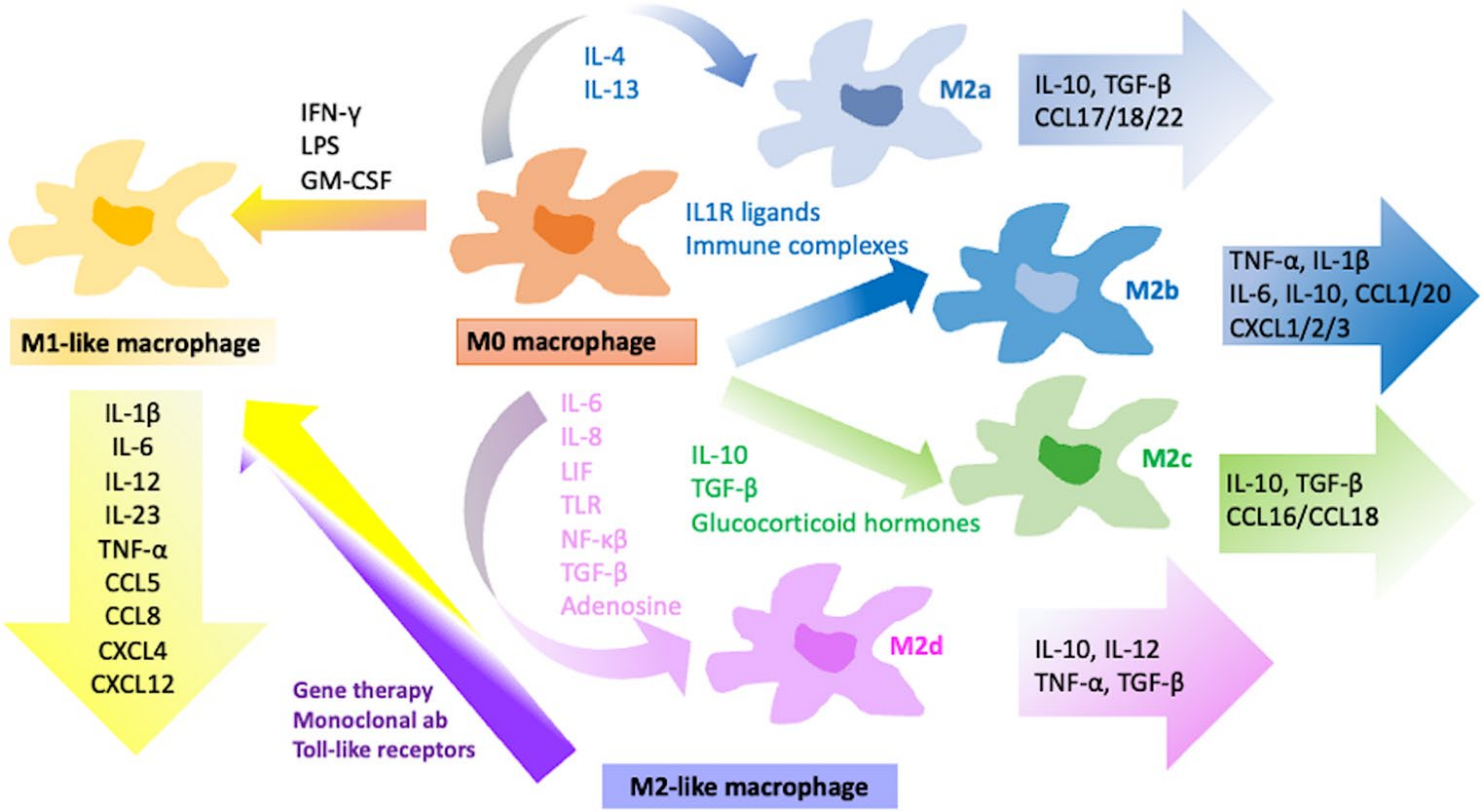
Macrophage polarization and stratification of M1 and M2 polarized macrophage subtypes with specific inducers and soluble factors

As evidenced by both cellular and molecular immunophenotypic studies, the role of TAMs in the TME is complex and multifaceted, and it can be either beneficial or detrimental to the tumor progression. This dichotomy stems from TAMs polarizing into two distinct functional subtypes: M1 and M2 macrophages (Fig. 2).

Macrophage polarization into M1 macrophages can occur through various signals (Fig. 2). Interferon-γ (IFN-γ) secretion by Type 1 T helper cells (T_h_1), NK cells and CD8^+^ T cells contact with lipopolysaccharides (LPS), and GM-CSF can all promote M1 polarization [34]. M1 macrophages are intrinsically pro-inflammatory, characterized by their increased ability to secrete pro-inflammatory cytokines for instance TNF, IL-1β, IL-12 and IL-18.

Metabolic functions of M1 macrophages are also developed to sustain their pro-inflammatory function, as M1 macrophages, compared to other subtypes, hinge on glycolysis and pentose phosphate pathway to maintain their energy needs [35].The presence of M1 macrophages is favorable in early stages of tumor development as they have shown to possess anti-tumor properties [36, 37].

M1 macrophages can attract CD8^+^ T lymphocytes and NK cells towards the TME by presenting antigens and secreting tumor-derived chemokines such as CXCL9, CXCL10, and CXCL11. Activated CD8^+^ T cells and NK cells in turn can secrete cytokines TNF-α, GM-CSF and IFN-γ, and chemokines CCL4, CCL5 and CCL23 to recruit even more immune cells to the TME to help suppress tumor growth [37].

## Alternatively activated macrophage (M2) polarization shaped by tumor niche

In healthy individuals, alternatively activated macrophages, namely M2 macrophages, undergo polarization in response to fungal and parasitic infections, presence of apoptotic and necrotic cells, and downstream signaling from MCSF, IL-4, IL-13, IL-10 and TGF-β. The M2 subtype can be characterized primarily by the expression of pro-repair proteins Arginase-1 and Ym1 as well as surface marker CD206. In cancer, however, the M2 subtype is achieved through IL-4 and IL-13 secreted by T helper 2 cells [38]. Depending on cytokines and signaling pathways involved in macrophage activation, M2 polarization can result in one of four subtypes: M2a, M2b, M2c and M2d macrophages [6] (Fig. 2).

M2 macrophages act differently from M1 macrophages and differ in general through exhibiting lower glycolytic activity, enhanced aerobic citric acid cycle (TCA cycle) and oxidative phosphorylation (OXPHOS) reactions, and overall immunosuppressive effect [39].

M2 subtypes can be distinguished from each other by their metabolic signatures in addition to the deep omic-based profiling analysis. According to an analysis based on differentially expressed proteins conducted by Li et al. M2a macrophages play a role in the functions of phospholipids in phagocytosis and retinoic acid signaling; M2b participates in the transport of amino acids across the plasma membrane; M2c is engaged in the control of neutrophil chemotaxis; and M2d involves in the somatic recombination of immunoglobulin gene segments [40]. Differences between M2 subtypes are not confined to these biological roles; omic studies of largely unexplored M2 subtypes will be directed by single cell analyses.

### M2a-type TAMs are related to type II inflammation

M2a macrophages assist in tissue reconstruction and remodeling, immune regulation, and defense against parasites. M2a phenotype is achieved through IL-4 and/or IL-13 cytokine signaling, which are typically produced by T helper 2 (Th2) cells. M2a macrophages can be characterized through their high expression of the surface markers CD206, CD163, and scavenger receptor-A (SR-A) [41].

M2a macrophages in healthy individuals release a variety of cytokines and chemokines, such as IL-10, TGF-, CCL17, CCL18, and CCL22, which helps regulate the immune response and promote tissue repair and remodeling (Fig. 2) [11]. M2a macrophages, on the other hand, have been associated with tumor development because they generate growth factors and cytokines such as VEGF and PDGF, which can promote angiogenesis. In addition to promoting tumor growth, M2a macrophages also participate in tumor invasion and metastasis through production of a variety of proteases, such as MMPs and cathepsins, that degrade the ECM and facilitate tumor cell invasion and migration [42]. Furthermore, M2a macrophages can produce anti-inflammatory cytokines and chemokines, such as TGF-β and IL-10 (Fig. 2), that inhibit the activation and proliferation of T cells and NK cells, which are critical for host’s anti-tumoral immune response.

**Fig. 3:**
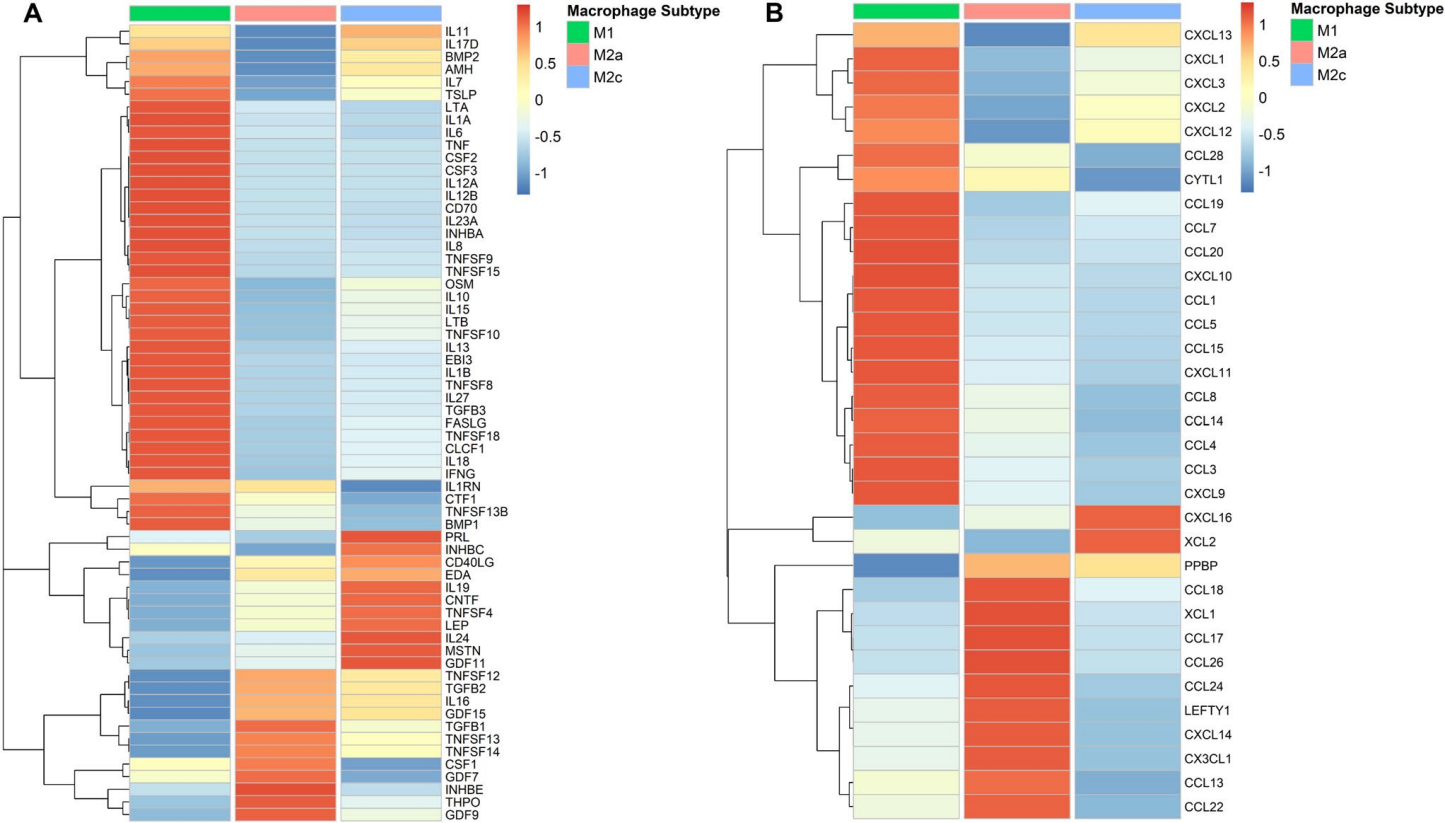
Comparative transcriptomic analysis of cytokine (**A**) and chemokine (**B**) gene expression profiles of classically polarized M1 and alternatively polarized M2a and M2c macrophages

Comparative analysis of cytokine (Fig. 3A) and chemokine (Fig. 3B) gene expression profiles between classically polarized M1, and alternatively polarized M2a and M2c macrophages were visualized in a heatmap. GEO dataset GSE227737 was used to analyze RNA-Seq data [43] of M1, M2a and M2c macrophages. Trimmed Means of M-Values (TMM) normalization was used to normalize the count values and edgeR package was used to determine differentially expressed genes across all groups. Genes with logarithmic fold change greater than 2 (logFC > 2) and false discovery rate smaller than 0.05 (FDR < 0.05) were considered to have statistically significant variance across groups. Kyoto Encyclopedia of Genes and Genomes (KEGG) Brite database was used to evaluate the gene expression of human cytokines and chemokines.

Although extensive studies are yet to be carried out in terms of how M2 subtypes differ in their metabolism, it has been reported that the M2a macrophages display the most distinct metabolic functions compared to the other M2 subtypes. M2a macrophages have reduced energy demands indicated by decreased aerobic TCA cycle and OXPHOS activity, and lower cellular uptake of glucose compared to other M2 subtypes, pointing towards a reduced level of glycolytic metabolism [44].

### An inhibitory effect on the immune response by the substantial factors released by M2b polarized macrophages

M2b subtype is acquired upon activation of immune complexes and TLR signaling, as well as IL1R activation. Upon receiving signals from either one of these sources, NF-κB p50 translocation into nucleus mediated by PI3K/Akt and MAPK signals is shown to be crucial in M2b polarization. In addition to NF-κB p50, IRF3, a transcription factor expressed in macrophages, is reported to be activated during M2b polarization [45]. Although M2b cells can secrete pro-inflammatory cytokines such as IL-1β, IL-6, TNF-α, CXCL1, CXCL2 and CXCL3, they are also found to be secreting significant amounts of IL-10 and low levels of IL-12 (Fig. 2) [45].

In addition to a plethora of pro- and anti- inflammatory cytokine secretion, CCL1 expression seems to be the hallmark of the M2b subtype of macrophages and essential for maintaining the M2b subtype [46]. CCL1 is a chemoattractant that recruits NK cells, immature B cells and DCs via interaction through cell surface CCR8. In M2b macrophages, though, autocrine production of CCL1 signaling helps cells survive without the need of exogenous growth factor. In tumor settings, this autocrine ability to proliferate helps M2b macrophages live longer than its counterparts and the prolonged presence of M2b macrophages have been reported to be linked with continuous inhibition of host anti-tumor immunity [47].

In contrast to M2a macrophages, M2b, M2c and M2d macrophages rely more on anaerobic glycolysis for ATP production than TCA cycle and OXPHOS. Furthermore, M2b macrophages can be distinguished from M2c and M2d in terms of TCA cycle intermediates such as enhanced intracellular glutamate, succinate and fumarate as well as increased extracellular lactate levels reflecting more active TCA cycle and glycolytic metabolism, respectively. Hence, supporting the concept that the M2b macrophages have the most ATP production amongst the M2 subtypes [44].

### M2c-specific genes are involved in phagocytosis, angiogenesis, and tumor niche modification through the scavenging of pro-inflammatory factors

M2c macrophage subtype is another anti-inflammatory M2 subtype that is induced by glucocorticoids or IL-10 dependent M-CSF signaling. M2c macrophages aid efferocytosis through phagocytosis of apoptotic cells [48]. They achieve this by having a high level of mer tyrosine kinase (MerTK) expression, which helps them identify apoptotic cells. In addition to elevated MerTK expression, they can also be characterized by comparatively high expression levels of CD14, CD163, CD169 and CD206, CCL16 and CCL18 [49, 50]. Furthermore, comparative transcriptomic analysis between macrophage subtypes has shown that the increased XCL2 and CXCL16 gene expression by M2c macrophages (Fig. 3B).

IL10 production by M2c macrophages is sustained by the production of Growth Arrest-Specific 6 (GAS6), which is a ligand for MerTK. As a result, the increase in IL10 production generates a positive loop in M2c macropahges [51]. In addition to GAS6, M2c macrophages were demonstrated to have a decreased phospholipid synthesis, which reduces the secretion metabolites required to produce pro-inflammatory cytokines such as IL1β and induces production of IL4 and IL13 [44].

In tumorigenic conditions, M2c macrophages act similarly to M2a macrophages as they both release anti-inflammatory cytokines for instance IL10 and TGF-β (Fig. 2), as well as induce tumor invasion and metastasis through expression of VEGF and MMPs. In addition to their chemokine secretion, M2c macrophages possess increased levels of chemokine receptors CCR2 and CCR5 whose expression is induced by IL-10 signaling. However, it has been proposed that these chemokine receptors, when found in M2c macrophages, act as functional decoy receptors which scavenge proinflammatory chemokines in the TME [52].

### M2d-type macrophages are differentiated by adenosine receptor agonists

Macrophage polarization is not a terminal fate for macrophages; in fact, polarized macrophages can switch from one subtype to another depending on the circumstances of their microenvironment. M2d macrophages are the prime example of this subtype switching as unlike other subtype mentioned above, M2d macrophages are derived from polarized M1 macrophages. Increasing levels of extracellular adenosine reported to be a key factor in M2d polarization; as M1 macrophages with high expression levels of adenosine receptors A_2A_R, respond to high extracellular adenosine levels by switching into anti-inflammatory and angiogenic M2d subtype of macrophages (Fig. 2) [53]. Adenosine production by apoptotic and necrotic cells can result in high extracellular adenosine levels. In addition to high extracellular adenosine levels, extracellular creatine, secreted by M2d macrophages can also induce M2 polarization and inhibit TLR expression to prevent inflammatory responsive niche in the TME. On the other hand, intracellular creatine concentration and uptake of creatine contribute to the maintenance of ATP homeostasis amidst phagocytosis [44].

M2 macrophage subtypes (M2a-d), while distinct, share similarities in their roles in tumor growth. M2 subtypes have characteristics that are mostly anti-inflammatory and pro-angiogenic, secreting growth factors (VEGF, PDGF) and matrix metalloproteinases (MMP2, MMP9) promoting tumor growth, metastasis, and invasion. Unlike their M1 counterparts, M2 macrophages secrete primarily immunosuppressive cytokines such as IL-4, IL-10 and IL-13 and are generally linked with tissue repair, cell proliferation and angiogenesis.

Thus, the M2 subpopulation of macrophages constitutes a much more heterogeneous group than M1 macrophages. Also, comparative transcriptomic analysis shows a remarkable distinction between M1 and M2 subtypes, and although similar in functional properties, M2 subtypes differ in their expression of cytokines and chemokines. (Fig. 3A-B).

## Current targeting strategies for M2 macrophages in cancer immunotherapy

Higher proportion of M2 cells in the TME has been linked with poor prognosis and reduced immunotherapy response in several cancer types including ovarian cancer [54], non-small cell lung cancer [55], multiple myeloma [56] and colorectal cancer [57]. Hence, targeting M2 macrophages has been an ongoing focus for improving immunotherapy outcomes.

In a recent study using adenoviruses engineered to express IL-2 and TNF-α that reportedly reduces the number of M2 macrophages among other immunosuppressive cells and increases the efficiency of anti-PD1 therapy [58]. Another way of hampering M2 polarization is targeting IL-4R in M2 macrophages. It has been reported that the inhibition of IL-4 signaling by using IL-4 binding peptides resulted in downregulation of M2 related markers and upregulation of M1 related genes [59].

In addition to cytokines, several metabolic pathways contribute to M2 polarization of macrophages. A recent study has shown that enhanced Zeb1 expression in THP-1 acute monocytic leukemia cell line resulted in increased expression of M2 markers such as CD206, Arg1 and IL-10, concluding that Zeb1 induced aerobic glycolysis contributes to M2 polarization of macrophages [60]. In addition, the Notch pathway has also been identified to be a key component of macrophage polarization, taking part in both M1-like and M2-like TAM generation [61]. Upon activation of the Notch signaling pathway, the resulting macrophage subtype reportedly depends on upstream ligand-receptor interaction. Macrophages, co-cultured with Delta Like Canonical Notch Ligand 4 (DDL4) expressing cells in vitro, have reported to have increased Neurogenic locus notch homolog protein 1 (NOTCH1) expression and a pro-inflammatory M1-like subtype and the blockage of DDL4 resulted in an M2-like subtype. However, further studies need to be performed to determine the extent to which the Notch signaling pathway modifies macrophage polarization in the TME [62].

## Immunosuppressive cells in cancer contributing pro-tumoral (M2) macrophage polarization

TAMs primarily play an M2-like tumor-promoting role in the TME and influence multiple malignant processes such as immune suppression, angiogenesis, and tumor dissemination. M2 macrophages can be regulated by other immune cells, such as Treg cells, MDSCs and B cells This review emphasizes M2 macrophage polarization as well as the impact of other immune/stromal cells on polarization within the immunosuppressive niche. (Fig. 1). A research study in the literature supports this notion that when M2a macrophages cultured with the preconditioned-Mesenchymal stem cells (preMSC-CM) revealed increased expression of M2b/M2c-specific markers, implying that the secretome of preMSCs supports the repolarization of M2a-like macrophages to M2b/M2c subtypes [63].

### Regulatory T cells (Tregs)

Malignant tumors with worse prognosis are related to elevated regulatory T cells and diminished M1 macrophages. M2 TAMs, as opposed to M1 macrophages, are more likely to encourage the recruitment of regulatory T cells to the TME [64–66].

There is a particular association between M2 macrophages and Tregs, a variety of solid tumors, as detected in colon cancer. High amounts of TGF-β, IL-10 and IL-32 are produced by Tregs, which further suppress the anti-tumor inflammatory response and trigger M2 macrophages to generate more cytokines and chemokines, allowing for the recruitment of more Tregs. Likewise, M2 macrophages and Tregs have been shown to have a synergistic impact in boosting ovarian cancer metastasis, tumor angiogenesis, and proliferation [4, 67–70]. The prevalence of M2 macrophages in Epstein-Barr virus-related Nasopharyngeal Carcinoma TME was associated with Treg, and both types of cells were related with tumor size and clinical stages [4]. In addition, co-infiltration of Tregs and M2 TAMs is significantly correlated with progression of premalignant lesions to oral squamous cell carcinoma as well [65].

The recruitment of CCR6^+^Foxp3^+^ regulatory T cells, a distinct effector/memory-like T cell subset, is dependent on the infiltration of TREM-1^+^ TAMs leading in immunosuppression within the hypoxic niche of hepatocellular carcinoma for resistance to anti-PD-L1 [71, 72]. Consequently, addressing the specific subtype of TAMs should not be underestimated in malignancies that acquire immunotherapy resistance.

TAMs can be controlled indirectly by Treg cells as well. The primary cytokine that blocks M2-like TAM is IFN-g. Tregs possess the ability to block the release of IFN- γ by CD8^+^ T cells, which prevents the activation of fatty acid production in immunosuppressive M2-like TAM mediated by sterol regulatory element binding protein 1 (SREBP1). In conclusion, metabolic stability, mitochondrial integrity, and survival rate of M2-like TAMs are selectively maintained by Treg cells in an indirect context [2, 73].

TAMs and Tregs tend to communicate in the opposite directions. Tregs can be recruited to TME by CCL1 secreting M2b macrophages [51], as well as TGF-β and IL-10 producing M2c macrophages, thus creating a loop that supports anti-inflammatory phenotype in TME maintained by both M2 TAMs and Tregs [74].

As a result, assessing Tregs and M2 macrophages combined will assist in predicting the cancer progression. Taking Treg/M2 TAM status into consideration will also contribute to immunotherapeutic approaches regarding the prognosis of the disease.

### Regulatory B cells (Bregs)

In addition to monocytes, which are their primary source, tumor-associated macrophages (TAM) can also develop from B-cell precursors. They differ from monocyte-derived TAM in inducing FoxP3^+^ Tregs in these macrophages, suppressing proliferation of T cells, and phagocytizing apoptotic cells more efficiently. Since they are derived from B cell precursors (pre-B and immature B cells into TAM), they are called B-MF [75] and contribute to escape mechanisms in cancer.

IL-10 released from B reg cells exerts anti-inflammatory effects in mice and humans and suppresses Th1 and Th17 responses. It leads to the production of CD4^+^CD25^+^FoxP3^+^ Treg cells and the suppression of macrophage and monocyte-derived inflammatory cytokines [76]. CD1d^hi^CD5^+^ Breg cells producing IL-10 diminish TNFα and NO production. It diminishes the expression of markers associated with monocyte activation. Even a small number of CD1d^hi^CD5^+^Breg causes antibody-mediated depletion of lymphoma cells due to the cytotoxic activity of monocytes and macrophages [77, 78]. Therefore, the contribution of Breg cells to macrophage polarization towards the M2 direction in different cancer types [76] should also be evaluated.

### Myeloid-derived suppressor cells (MDSCs)

Myeloid-derived suppressor cells (MDSC) are detected in many cancer patients as well as in vivo cancer models and they serve to suppress the immune system by preventing CD4 and CD8 T cell activation. Communication between macrophages and MDSCs further subverts tumor immunity by enhancing production of IL-10 by MDSC and diminishing IL-10 production derived from macrophages in spontaneously metastatic 4T1 mouse mammary cancer [78]. The elevated generation of IL-10 originated from MDSCs also reshapes helper T cell differentiation towards the Th2 phenotype, impacting cytotoxic T lymphocyte development.

Th2 cells also generate a large amount of IL-4, which contributes to the formation of TAMs, as well. Crosstalk with MDSC may also influence macrophage MHC II expression, while IL10 has been shown to boost the expression of March 1, a ubiquitin ligase family member necessary for the ubiquitination of the cytoplasmic tail of MHC II molecules in monocytes [79, 80]. This indicates that MDSC cells potentially influence antigen-presenting capabilities of TAMs. Levels of MDSCs and CD163^+^ M2 macrophages are found to be elevated correlatively in NSCLC patients after they receive ionizing radiation therapy, pointing towards potential biomarkers during NSCLC diagnosis and favorable therapeutic response [81].

In a mouse breast cancer model, breast tumor initiation is linked to the shift of tumor infiltrating MDSCs to TAMs driven by the hypoxic TME [82]. HIF1α is a key regulator of MDSC development and function in the TME. HIF1 supports the differentiation of M-MDSCs into tumor-associated macrophages via a process involving CD45 tyrosine phosphatase activity and STAT3 downregulation [83–85].

### Alternatively activated (N2) neutrophils

TAMs and tumor associated neutrophils (TANs) are polarized by the TME to become pro-tumoral and favor tumor growth and development, invasiveness as well as matrix remodeling, angiogenesis, and metastasis, while blocking anti-tumoral immune surveillance [86]. The findings of the KrasLSL-G12D/+; p53fl/fl mouse model has demonstrated that the spleen mobilizes immature myeloid cells, and that these cells augment TAM and TAN responses compromised in the tumor progression. With regard to this, a high number of TAM and TAN precursors physically migrated from the spleen to the tumor stroma, and CCR2 signaling is involved in the recruitment of tumor-promoting spleen-derived TAMs [87]. Further research into the mechanism behind all this mutual exclusion is warranted.

Triple negative mouse breast cancer models have demonstrated that the tumors did not attract TANs and TAMs uniformly. Even though they are the same breast cancer subtype, they might be immuno-subtyped as neutrophil-enriched (NES, CD11b^+^ Ly6C ^mid^ Ly6G^+^) or macrophage-enriched (MES, CD11b^+^ Ly6G^−^ Ly6C^−^ F4/80^+^) based on their predisposition for recruiting TANs and TAMs which shown as mutually exclusive. TAM was triggered when TAN was depleted, or vice versa.

## Conclusion and future perspective

The molecular goals have been to convert M2-polarized macrophages into M1-like macrophages via manipulating signaling pathway ligands (such as TLR), monoclonal antibodies, and miRNA/siRNA-based gene therapy [88]. Table 1 provides a summary of completed/ongoing clinical studies evaluating macrophage polarization in multiple types of solid cancer.

**Table 1:** The clinical trials focusing macrophage polarization in different types of solid cancer

Purpose of the study	Cancer type	Study status	Clinical trial identification
The evaluation of correlation between M1/M2 phenotype and clinical outcome.	Lung cancer	Unknown	**NCT00690261**
Investigaton of correlation between M1/M2 macrophage polarization and [18 F] DPA-714 PET/CT binding.	Triple Negative Breast	Completed	**NCT04320030**
Characterization of TAMs under combined effect of metformin and durvalumab.	Head and Neck Squamous Cell Carcinoma Oral Cavity Squamous Cell Carcinoma Oropharyngeal Squamous Cell Carcinoma	Active	**NCT03618654**

As a result of the extensive crosstalk between tumor-associated macrophages (M2-like macrophages) associated with poor prognosis in the tumor niche, personalized multi-parametric therapeutic strategies should be taken into consideration and collectively analyzed. Thus, the remarkable improvement can be achieved relevant to therapeutic strategies, especially in immunotherapy approaches.

Combined therapeutic regimens including conventional, and immunotherapy should also be rigorously developed based on tumor niche characteristics to provide synergistic action while avoiding major side effects. Besides, insightful regulatory mechanisms between M2 macrophages and other immunosuppressive immune cells will pay the way for the cancer drug discovery in terms of targeting tumor microenvironment and insightful immunotherapy approaches.

## Data Availability

GEO dataset GSE227737 was used to analyze RNA-Seq data and stated within the manuscript.

## Abbreviations

CSC: Cancer stem cells
CSF: Colony stimulating factor
DC: Dendritic cells
ECM: Extracellular matrix
EMT: Epithelial-mesenchymal transition
FDR: False Discovery Rate
FGF: Fibroblast growth factor
GAS6: Growth arrest-specific 6
HIF: Hypoxia inducible factor
IFN-γ: Interferon gamma
IL: Interleukin
LogFC: Logarithmic Fold Change
LPS: Lipopolysaccharides
MDSC: Myeloid-derived suppressor cells
MMP: Matrix metalloproteinase
MSC: Mesenchymal stem cells
NK: Natural killer cells
NO: Nitric oxide
OXPHOS: Oxidative phosphorylation
PDGF: Platelet derived growth factor
ROS: Reactive oxygen species
TAM: Tumor associated macrophage
TAN: Tumor associated neutrophils
TCA Cycle: The citric acid cycle
TGF-β: Transforming growth factor beta
TLR: Toll like receptor
TME: Tumor microenvironment
TMM: Trimmed Means of M-Values
TNF-α: Tumor necrosis factor alpha
VEGF: Vascular endothelial growth factor

## References

[1] Bożyk A, Wojas-Krawczyk K, Krawczyk P, Milanowski J. Tumor Microenvironment—A Short Review of Cellular and Interaction Diversity. Biology. 2022;11:929. 35741450 10.3390/biology11060929 PMC9220289

[2] Pan Y, Yu Y, Wang X, Zhang T. Tumor-Associated macrophages in Tumor Immunity. Front Immunol. 2020;11:583084. 33365025 10.3389/fimmu.2020.583084 PMC7751482

[3] Yao Y, Xu X-H, Jin L. Macrophage polarization in physiological and pathological pregnancy. Front Immunol. 2019;10:792. 31037072 10.3389/fimmu.2019.00792 PMC6476302

[4] Aliyah S, Ardiyan Y, Mardhiyah I, Herdini camelia, Dwianingsih E, Aning S, et al. The distribution of M2 macrophage and Treg in Nasopharyngeal Carcinoma Tumor Tissue and the correlation with TNM Status and Clinical Stage. Asian Pac J Cancer Prev. 2021;22:3447–53. 34837898 10.31557/APJCP.2021.22.11.3447 PMC9068172

[5] He H, Xu J, Warren CM, Duan D, Li X, Wu L, et al. Endothelial cells provide an instructive niche for the differentiation and functional polarization of M2-like macrophages. Blood. 2012;120:3152–62. 22919031 10.1182/blood-2012-04-422758 PMC3471522

[6] Yunna C, Mengru H, Lei W, Weidong C. Macrophage M1/M2 polarization. Eur J Pharmacol. 2020;877:173090. 32234529 10.1016/j.ejphar.2020.173090

[7] Wang M, Zhao J, Zhang L, Wei F, Lian Y, Wu Y, et al. Role of tumor microenvironment in tumorigenesis. J Cancer. 2017;8:761–73. 28382138 10.7150/jca.17648 PMC5381164

[8] Qian B-Z, Pollard JW. Macrophage diversity enhances Tumor Progression and Metastasis. Cell. 2010;141:39–51. 20371344 10.1016/j.cell.2010.03.014 PMC4994190

[9] Wu H, Xu J-B, He Y-L, Peng J-J, Zhang X-H, Chen C-Q, et al. Tumor-associated macrophages promote angiogenesis and lymphangiogenesis of gastric cancer. J Surg Oncol. 2012;106:462–8. 22488237 10.1002/jso.23110

[10] Yuan Z-Y, Luo R-Z, Peng R-J, Wang S-S, Xue C. High infiltration of tumor-associated macrophages in triple-negative breast cancer is associated with a higher risk of distant metastasis. OncoTargets Ther. 2014;1475. 10.2147/OTT.S61838 PMC4149399 25187727

[11] Wang Q, Sudan K, Schmoeckel E, Kost BP, Kuhn C, Vattai A, et al. CCL22-Polarized TAMs to M2a macrophages in Cervical Cancer in Vitro Model. Cells. 2022;11:2027. 35805111 10.3390/cells11132027 PMC9265611

[12] Semenza GL, Oxygen Sensing. Hypoxia-inducible factors, and Disease Pathophysiology. Annu Rev Pathol Mech Dis. 2014;9:47–71. 10.1146/annurev-pathol-012513-104720 23937437

[13] Takeda N, O’Dea EL, Doedens A, Kim J, Weidemann A, Stockmann C, et al. Differential activation and antagonistic function of HIF-α isoforms in macrophages are essential for NO homeostasis. Genes Dev. 2010;24:491–501. 20194441 10.1101/gad.1881410 PMC2827844

[14] Lewis CE, Pollard JW. Distinct role of macrophages in different tumor microenvironments. Cancer Res. 2006;66:605–12. 16423985 10.1158/0008-5472.CAN-05-4005

[15] Su S, Liu Q, Chen J, Chen J, Chen F, He C, et al. A positive Feedback Loop between Mesenchymal-Like Cancer cells and macrophages is essential to breast Cancer metastasis. Cancer Cell. 2014;25:605–20. 24823638 10.1016/j.ccr.2014.03.021

[16] Shen W, Tao G, Zhang Y, Cai B, Sun J, Tian Z. TGF-β in pancreatic cancer initiation and progression: two sides of the same coin. Cell Biosci. 2017;7:39. 28794854 10.1186/s13578-017-0168-0 PMC5545849

[17] Horiguchi K, Sakamoto K, Koinuma D, Semba K, Inoue A, Inoue S, et al. TGF-β drives epithelial-mesenchymal transition through δEF1-mediated downregulation of ESRP. Oncogene. 2012;31:3190–201. 22037216 10.1038/onc.2011.493 PMC3391666

[18] Cruceriu D, Baldasici O, Balacescu O, Berindan-Neagoe I. The dual role of tumor necrosis factor-alpha (TNF-α) in breast cancer: molecular insights and therapeutic approaches. Cell Oncol. 2020;43:1–18. 10.1007/s13402-019-00489-1 PMC12990688 31900901

[19] Dong W, Sun S, Cao X, Cui Y, Chen A, Li X, et al. Exposure to TNF-α combined with TGF-β induces carcinogenesis in vitro via NF-κB/Twist axis. Oncol Rep. 2017;37:1873–82. 28098875 10.3892/or.2017.5369

[20] Che D, Zhang S, Jing Z, Shang L, Jin S, Liu F, et al. Macrophages induce EMT to promote invasion of lung cancer cells through the IL-6-mediated COX-2/PGE 2 /β-catenin signalling pathway. Mol Immunol. 2017;90:197–210. 28837884 10.1016/j.molimm.2017.06.018

[21] Deng F, Weng Y, Li X, Wang T, Fan M, Shi Q. Overexpression of IL-8 promotes cell migration via PI3K-Akt signaling pathway and EMT in triple-negative breast cancer. Pathol - Res Pract. 2021;223:152824. 34000674 10.1016/j.prp.2020.152824

[22] Liu C-Y, Xu J-Y, Shi X-Y, Huang W, Ruan T-Y, Xie P, et al. M2-polarized tumor-associated macrophages promoted epithelial–mesenchymal transition in pancreatic cancer cells, partially through TLR4/IL-10 signaling pathway. Lab Invest. 2013;93:844–54. 23752129 10.1038/labinvest.2013.69

[23] Borovski T, De Sousa E, Melo F, Vermeulen L, Medema JP. Cancer Stem Cell Niche: the place to be. Cancer Res. 2011;71:634–9. 21266356 10.1158/0008-5472.CAN-10-3220

[24] Jinushi M, Chiba S, Yoshiyama H, Masutomi K, Kinoshita I, Dosaka-Akita H, et al. Tumor-associated macrophages regulate tumorigenicity and anticancer drug responses of cancer stem/initiating cells. Proc Natl Acad Sci. 2011;108:12425–30. 21746895 10.1073/pnas.1106645108 PMC3145680

[25] Zhang B, Ye H, Ren X, Zheng S, Zhou Q, Chen C, et al. Macrophage-expressed CD51 promotes cancer stem cell properties via the TGF-β1/smad2/3 axis in pancreatic cancer. Cancer Lett. 2019;459:204–15. 31199988 10.1016/j.canlet.2019.06.005

[26] Liguori M, Digifico E, Vacchini A, Avigni R, Colombo FS, Borroni EM, et al. The soluble glycoprotein NMB (GPNMB) produced by macrophages induces cancer stemness and metastasis via CD44 and IL-33. Cell Mol Immunol. 2021;18:711–22. 32728200 10.1038/s41423-020-0501-0 PMC8027814

[27] Lin L, Li Y, Liu M, Li Q, Liu Q, Li R. The Interleukin-33/ST2 axis promotes glioma mesenchymal transition, stemness and TMZ resistance via JNK activation. Aging. 2020;12:1685–703. 32003751 10.18632/aging.102707 PMC7053587

[28] Ma P-F, Gao C-C, Yi J, Zhao J-L, Liang S-Q, Zhao Y, et al. Cytotherapy with M1-polarized macrophages ameliorates liver fibrosis by modulating immune microenvironment in mice. J Hepatol. 2017;67:770–9. 28596109 10.1016/j.jhep.2017.05.022

[29] Li H, Shi B. Tolerogenic dendritic cells and their applications in transplantation. Cell Mol Immunol. 2015;12:24–30. 25109681 10.1038/cmi.2014.52 PMC4654373

[30] Solinas G, Schiarea S, Liguori M, Fabbri M, Pesce S, Zammataro L, et al. Tumor-conditioned macrophages Secrete Migration-stimulating factor: a new marker for M2-Polarization, influencing Tumor Cell Motility. J Immunol. 2010;185:642–52. 20530259 10.4049/jimmunol.1000413

[31] Otsuji M, Kimura Y, Aoe T, Okamoto Y, Saito T. Oxidative stress by tumor-derived macrophages suppresses the expression of CD3 ζ chain of T-cell receptor complex and antigen-specific T-cell responses. Proc Natl Acad Sci. 1996;93:13119–24. 8917554 10.1073/pnas.93.23.13119 PMC24056

[32] Yin C, Han Q, Xu D, Zheng B, Zhao X, Zhang J. SALL4-mediated upregulation of exosomal miR-146a-5p drives T-cell exhaustion by M2 tumor-associated macrophages in HCC. OncoImmunology. 2019;8:e1601479. 10.1080/2162402X.2019.1601479 PMC6527304 31143524

[33] del Rio M-L, Buhler L, Gibbons C, Tian J, Rodriguez-Barbosa J-I. PD-1/PD-L1, PD-1/PD-L2, and other co-inhibitory signaling pathways in transplantation. Transpl Int off J Eur Soc Organ Transpl. 2008;21:1015–28. 10.1111/j.1432-2277.2008.00726.x 18662368

[34] Andrade MR, Amaral EP, Ribeiro SC, Almeida FM, Peres TV, Lanes V, et al. Pathogenic Mycobacterium bovis strains differ in their ability to modulate the proinflammatory activation phenotype of macrophages. BMC Microbiol. 2012;12:166. 22863292 10.1186/1471-2180-12-166 PMC3478980

[35] Kiseleva V, Vishnyakova P, Elchaninov A, Fatkhudinov T, Sukhikh G. Biochemical and molecular inducers and modulators of M2 macrophage polarization in clinical perspective. Int Immunopharmacol. 2023;122:110583. 37423155 10.1016/j.intimp.2023.110583

[36] Bernsmeier C, van der Merwe S, Périanin A. Innate immune cells in cirrhosis. J Hepatol. 2020;73:186–201. 32240716 10.1016/j.jhep.2020.03.027

[37] Dungan LS, McGuinness NC, Boon L, Lynch MA, Mills KHG. Innate IFN-γ promotes development of experimental autoimmune encephalomyelitis: a role for NK cells and M1 macrophages. Eur J Immunol. 2014;44:2903–17. 25056715 10.1002/eji.201444612

[38] Anderson NR, Minutolo NG, Gill S, Klichinsky M. Macrophage-based approaches for Cancer Immunotherapy. Cancer Res. 2021;81:1201–8. 33203697 10.1158/0008-5472.CAN-20-2990

[39] Viola A, Munari F, Sánchez-Rodríguez R, Scolaro T, Castegna A. The metabolic signature of macrophage responses. Front Immunol. 2019;10:1462. 31333642 10.3389/fimmu.2019.01462 PMC6618143

[40] Li P, Ma C, Li J, You S, Dang L, Wu J, et al. Proteomic characterization of four subtypes of M2 macrophages derived from human THP-1 cells. J Zhejiang Univ-Sci B. 2022;23:407–22. 35557041 10.1631/jzus.B2100930 PMC9110321

[41] Pepe G, Calderazzi G, De Maglie M, Villa AM, Vegeto E. Heterogeneous induction of microglia M2a phenotype by central administration of interleukin-4. J Neuroinflammation. 2014;11:211. 25551794 10.1186/s12974-014-0211-6 PMC4302702

[42] Mantovani A, Biswas SK, Galdiero MR, Sica A, Locati M. Macrophage plasticity and polarization in tissue repair and remodelling: macrophage plasticity and polarization in tissue repair and remodelling. J Pathol. 2013;229:176–85. 23096265 10.1002/path.4133

[43] Gunalp S, Helvaci DG, Oner A, Bursalı A, Conforte A, Güner H et al. TRAIL promotes the polarization of human macrophages toward a proinflammatory M1 phenotype and is associated with increased survival in cancer patients with high tumor macrophage content. Front Immunol [Internet]. 2023;14. https://www.frontiersin.org/articles/10.3389/fimmu.2023.1209249. 10.3389/fimmu.2023.1209249 PMC10551148 37809073

[44] Fuchs AL, Costello SM, Schiller SM, Tripet BP, Copié V. Primary human M2 macrophage subtypes are distinguishable by Aqueous Metabolite profiles. Int J Mol Sci. 2024;25:2407. 38397084 10.3390/ijms25042407 PMC10889249

[45] Li Y, Cai L, Wang H, Wu P, Gu W, Chen Y, et al. Pleiotropic regulation of macrophage polarization and tumorigenesis by formyl peptide receptor-2. Oncogene. 2011;30:3887–99. 21499310 10.1038/onc.2011.112

[46] Asai A, Nakamura K, Kobayashi M, Herndon DN, Suzuki F. CCL1 released from M2b macrophages is essentially required for the maintenance of their properties. J Leukoc Biol. 2012;92:859–67. 22730547 10.1189/jlb.0212107

[47] Asai A, Tsuchimoto Y, Ohama H, Fukunishi S, Tsuda Y, Kobayashi M, et al. Host antitumor resistance improved by the macrophage polarization in a chimera model of patients with HCC. OncoImmunology. 2017;6:e1299301. 28507807 10.1080/2162402X.2017.1299301 PMC5414886

[48] Lai Y-S, Putra RBDS, Aui S-P, Chang K-T. M2C polarization by Baicalin enhances efferocytosis via Upregulation of MERTK receptor. Am J Chin Med. 2018;46:1899–914. 30518232 10.1142/S0192415X18500957

[49] Cappello P, Caorsi C, Bosticardo M, De Angelis S, Novelli F, Forni G, et al. CCL16/LEC powerfully triggers effector and antigen-presenting functions of macrophages and enhances T cell cytotoxicity. J Leukoc Biol. 2004;75:135–42. 14525962 10.1189/jlb.0403146

[50] Zizzo G, Hilliard BA, Monestier M, Cohen PL. Efficient clearance of early apoptotic cells by human macrophages requires M2c polarization and MerTK induction. J Immunol. 2012;189:3508–20. 22942426 10.4049/jimmunol.1200662 PMC3465703

[51] Zhang Q, Sioud M. Tumor-Associated Macrophage subsets: shaping polarization and targeting. Int J Mol Sci. 2023;24:7493. 37108657 10.3390/ijms24087493 PMC10138703

[52] Mantovani A, Sica A, Sozzani S, Allavena P, Vecchi A, Locati M. The chemokine system in diverse forms of macrophage activation and polarization. Trends Immunol. 2004;25:677–86. 15530839 10.1016/j.it.2004.09.015

[53] Ferrante CJ, Pinhal-Enfield G, Elson G, Cronstein BN, Hasko G, Outram S, et al. The Adenosine-Dependent Angiogenic switch of macrophages to an M2-Like phenotype is Independent of Interleukin-4 receptor alpha (IL-4Rα) signaling. Inflammation. 2013;36:921–31. 23504259 10.1007/s10753-013-9621-3 PMC3710311

[54] Macciò A, Gramignano G, Cherchi MC, Tanca L, Melis L, Madeddu C. Role of M1-polarized tumor-associated macrophages in the prognosis of advanced ovarian cancer patients. Sci Rep. 2020;10:6096. 32269279 10.1038/s41598-020-63276-1 PMC7142107

[55] Sumitomo R, Hirai T, Fujita M, Murakami H, Otake Y, Huang C. M2 tumor–associated macrophages promote tumor progression in non–small–cell lung cancer. Exp Ther Med. 2019;18:4490–8. 31777551 10.3892/etm.2019.8068 PMC6862535

[56] Chen X, Chen J, Zhang W, Sun R, Liu T, Zheng Y et al. Prognostic value of diametrically polarized tumor-associated macrophages in multiple myeloma. Oncotarget Vol 8 No 68 [Internet]. 2017 [cited 2017 Jan 1]; https://www.oncotarget.com/article/22340/text/. 10.18632/oncotarget.22340 PMC5762541 29348856

[57] Herrera M, Herrera A, Domínguez G, Silva J, García V, García JM, et al. Cancer-associated fibroblast and M2 macrophage markers together predict outcome in colorectal cancer patients. Cancer Sci. 2013;104:437–44. 23298232 10.1111/cas.12096 PMC7657228

[58] Cervera-Carrascon V, Quixabeira DCA, Santos JM, Havunen R, Milenova I, Verhoeff J et al. Adenovirus Armed With TNFa and IL2 Added to aPD-1 Regimen Mediates Antitumor Efficacy in Tumors Refractory to aPD-1. Front Immunol [Internet]. 2021;12. https://www.frontiersin.org/articles/10.3389/fimmu.2021.706517. 10.3389/fimmu.2021.706517 PMC8343222 34367166

[59] Gunassekaran GR, Poongkavithai Vadevoo SM, Baek M-C, Lee B. M1 macrophage exosomes engineered to foster M1 polarization and target the IL-4 receptor inhibit tumor growth by reprogramming tumor-associated macrophages into M1-like macrophages. Biomaterials. 2021;278:121137. 34560422 10.1016/j.biomaterials.2021.121137

[60] Jiang H, Wei H, Wang H, Wang Z, Li J, Ou Y, et al. Zeb1-induced metabolic reprogramming of glycolysis is essential for macrophage polarization in breast cancer. Cell Death Dis. 2022;13:206. 35246504 10.1038/s41419-022-04632-z PMC8897397

[61] Palaga T, Wongchana W, Kueanjinda P. Notch Signaling in macrophages in the Context of Cancer Immunity. Front Immunol. 2018;9:652. 29686671 10.3389/fimmu.2018.00652 PMC5900058

[62] Liu H, Wang J, Zhang M, Xuan Q, Wang Z, Lian X, et al. Jagged1 promotes aromatase inhibitor resistance by modulating tumor-associated macrophage differentiation in breast cancer patients. Breast Cancer Res Treat. 2017;166:95–107. 28730338 10.1007/s10549-017-4394-2

[63] Holthaus M, Santhakumar N, Wahlers T, Paunel-Görgülü A. The secretome of preconditioned mesenchymal stem cells drives polarization and reprogramming of M2a macrophages toward an IL-10-Producing phenotype. Int J Mol Sci. 2022;23:4104. 35456922 10.3390/ijms23084104 PMC9024470

[64] Davidsson S, Fiorentino M, Giunchi F, Eriksson M, Erlandsson A, Sundqvist P, et al. Infiltration of M2 Macrophages and Regulatory T Cells Plays a role in recurrence of renal cell carcinoma. Eur Urol Open Sci. 2020;20:62–71. 34337459 10.1016/j.euros.2020.06.003 PMC8317874

[65] Kouketsu A, Sato I, Oikawa M, Shimizu Y, Saito H, Tashiro K, et al. Regulatory T cells and M2-polarized tumour-associated macrophages are associated with the oncogenesis and progression of oral squamous cell carcinoma. Int J Oral Maxillofac Surg. 2019;48:1279–88. 31053518 10.1016/j.ijom.2019.04.004

[66] Sun W, Wei F-Q, Li W-J, Wei J-W, Zhong H, Wen Y-H, et al. A positive-feedback loop between tumour infiltrating activated Treg cells and type 2-skewed macrophages is essential for progression of laryngeal squamous cell carcinoma. Br J Cancer. 2017;117:1631–43. 28949956 10.1038/bjc.2017.329 PMC5729431

[67] Cao Q, Wang Y, Zheng D, Sun Y, Wang Y, Lee VWS, et al. IL-10/TGF-β–Modified Macrophages Induce Regulatory T Cells and protect against Adriamycin Nephrosis. J Am Soc Nephrol. 2010;21:933–42. 20299353 10.1681/ASN.2009060592 PMC2900959

[68] Chen Y, Gao Y, Ma X, Wang Y, Liu J, Yang C, et al. A study on the correlation between M2 macrophages and regulatory T cells in the progression of colorectal cancer. Int J Biol Markers. 2022;37:412–20. 36285512 10.1177/03936155221132572

[69] Ris MM, Deitrich RA, Von Wartburg JP. Inhibition of aldehyde reductase isoenzymes in human and rat brain. Biochem Pharmacol. 1975;24:1865–9. 18 10.1016/0006-2952(75)90405-0

[70] Tiemessen MM, Jagger AL, Evans HG, van Herwijnen MJC, John S, Taams LS. CD4 ^+^ CD25 ^+^ Foxp3 ^+^ regulatory T cells induce alternative activation of human monocytes/macrophages. Proc Natl Acad Sci. 2007;104:19446–51. 18042719 10.1073/pnas.0706832104 PMC2148309

[71] Wu Q, Zhou W, Yin S, Zhou Y, Chen T, Qian J, et al. Blocking triggering receptor expressed on myeloid cells-1‐Positive Tumor‐Associated macrophages Induced by Hypoxia reverses immunosuppression and anti‐programmed cell death Ligand 1 resistance in Liver Cancer. Hepatology. 2019;70:198–214. 30810243 10.1002/hep.30593 PMC6618281

[72] Yu S, Wang Y, Hou J, Li W, Wang X, Xiang L et al. E Wang editor 2020 Tumor-infiltrating immune cells in hepatocellular carcinoma: Tregs is correlated with poor overall survival. PLoS ONE 15 e0231003. 32240238 10.1371/journal.pone.0231003 PMC7117689

[73] Liu C, Chikina M, Deshpande R, Menk AV, Wang T, Tabib T, et al. Treg cells promote the SREBP1-Dependent metabolic fitness of Tumor-promoting macrophages via repression of CD8 + T cell-derived Interferon-γ. Immunity. 2019;51:381–e3976. 31350177 10.1016/j.immuni.2019.06.017 PMC6703933

[74] Vidyarthi A, Agnihotri T, Khan N, Singh S, Tewari MK, Radotra BD, et al. Predominance of M2 macrophages in gliomas leads to the suppression of local and systemic immunity. Cancer Immunol Immunother. 2019;68:1995–2004. 31690954 10.1007/s00262-019-02423-8 PMC11028103

[75] Chen C, Park B, Ragonnaud E, Bodogai M, Wang X, Zong L, et al. Cancer co-opts differentiation of B-cell precursors into macrophage-like cells. Nat Commun. 2022;13:5376. 36104343 10.1038/s41467-022-33117-y PMC9474882

[76] Schwartz M, Zhang Y, Rosenblatt JD. B cell regulation of the anti-tumor response and role in carcinogenesis. J Immunother Cancer. 2016;4:40. 27437104 10.1186/s40425-016-0145-x PMC4950763

[77] Horikawa M, Minard-Colin V, Matsushita T, Tedder TF. Regulatory B cell production of IL-10 inhibits lymphoma depletion during CD20 immunotherapy in mice. J Clin Invest. 2011;121:4268–80. 22019587 10.1172/JCI59266 PMC3204847

[78] Sinha P, Clements VK, Bunt SK, Albelda SM, Ostrand-Rosenberg S. Cross-talk between myeloid-derived suppressor cells and macrophages subverts Tumor immunity toward a type 2 response. J Immunol. 2007;179:977–83. 17617589 10.4049/jimmunol.179.2.977

[79] DeNardo DG, Barreto JB, Andreu P, Vasquez L, Tawfik D, Kolhatkar N, et al. CD4 + T cells regulate pulmonary metastasis of mammary carcinomas by enhancing Protumor properties of macrophages. Cancer Cell. 2009;16:91–102. 19647220 10.1016/j.ccr.2009.06.018 PMC2778576

[80] Thibodeau J, Bourgeois-Daigneault M-C, Huppé G, Tremblay J, Aumont A, Houde M, et al. Interleukin-10-induced MARCH1 mediates intracellular sequestration of MHC class II in monocytes. Eur J Immunol. 2008;38:1225–30. 18389477 10.1002/eji.200737902 PMC2759377

[81] Lv M, Zhuang X, Shao S, Li X, Cheng Y, Wu D et al. Myeloid-Derived Suppressor Cells and CD68 + CD163 + M2-Like Macrophages as Therapeutic Response Biomarkers Are Associated with Plasma Inflammatory Cytokines: A Preliminary Study for Non-Small Cell Lung Cancer Patients in Radiotherapy. Ortega E, editor. J Immunol Res. 2022;2022:1–16. 10.1155/2022/3621496 PMC9345704 35928634

[82] Fang Z, Wen C, Chen X, Yin R, Zhang C, Wang X, et al. Myeloid-derived suppressor cell and macrophage exert distinct angiogenic and immunosuppressive effects in breast cancer. Oncotarget. 2017;8:54173–86. 28903332 10.18632/oncotarget.17013 PMC5589571

[83] Corzo CA, Condamine T, Lu L, Cotter MJ, Youn J-I, Cheng P, et al. HIF-1α regulates function and differentiation of myeloid-derived suppressor cells in the tumor microenvironment. J Exp Med. 2010;207:2439–53. 20876310 10.1084/jem.20100587 PMC2964584

[84] Kumar V, Cheng P, Condamine T, Mony S, Languino LR, McCaffrey JC, et al. CD45 Phosphatase inhibits STAT3 transcription factor activity in myeloid cells and promotes Tumor-Associated Macrophage differentiation. Immunity. 2016;44:303–15. 26885857 10.1016/j.immuni.2016.01.014 PMC4759655

[85] Liu G, Bi Y, Shen B, Yang H, Zhang Y, Wang X, et al. SIRT1 limits the function and fate of myeloid-derived suppressor cells in tumors by orchestrating HIF-1 α –Dependent glycolysis. Cancer Res. 2014;74:727–37. 24351289 10.1158/0008-5472.CAN-13-2584

[86] Wu L, Zhang XH-F. Tumor-Associated neutrophils and macrophages—heterogenous but not chaotic. Front Immunol. 2020;11:553967. 33343560 10.3389/fimmu.2020.553967 PMC7738476

[87] Cortez-Retamozo V, Etzrodt M, Newton A, Rauch PJ, Chudnovskiy A, Berger C, et al. Origins of tumor-associated macrophages and neutrophils. Proc Natl Acad Sci. 2012;109:2491–6. 22308361 10.1073/pnas.1113744109 PMC3289379

[88] Poh AR, Ernst M. Targeting macrophages in Cancer: from bench to Bedside. Front Oncol. 2018;8:49. 29594035 10.3389/fonc.2018.00049 PMC5858529

